# HECT-type ubiquitin ligases: Emerging principles in the era of full-length structures

**DOI:** 10.1016/j.jbc.2026.111440

**Published:** 2026-04-09

**Authors:** Thornton J. Fokkens, Blanca Baños-Jaime, Sonja Lorenz

**Affiliations:** Research Group “Ubiquitin Signaling Specificity”, Max Planck Institute for Multidisciplinary Sciences, Göttingen, Germany

**Keywords:** cryo electron microscopy, E3, enzyme mechanism, posttranslational modification, substrate recognition, ubiquitination

## Abstract

Ubiquitin coordinates a complex network of cellular pathways through covalent modification of substrates. Specificity in substrate recognition and modification choice is largely conferred by ubiquitin ligases (E3s), a highly diversified enzyme family comprising 672 members in human cells. Among these, 28 belong to the homologous to E6AP C-terminus (HECT) family, whose distinctive structural features and functional specializations have remained incompletely understood. While the catalytic principles of the defining C-terminal HECT domain are well established, the manner in which this domain is embedded and regulated within full-length enzyme contexts long remained elusive. Over the past 5 years, a series of cryogenic electron microscopy studies have yielded unprecedented insight into the overall architectures, regulation modes, as well as linkage and substrate specificities of full-length HECT-type ligases. Here, we synthesize these advances to provide an up-to-date structural framework for HECT E3 mechanisms and highlight key questions for future investigation, including implications for small-molecule discovery.

Ubiquitination is a versatile posttranslational protein modification that dynamically modifies over 50,000 substrate sites in human cells, modulating all aspects of protein function (1). Moreover, ubiquitin can be conjugated to sugars, lipids and nucleotides, expanding its signaling scope beyond protein substrates (2). Ubiquitination is driven by a cascade of ubiquitin-activating enzymes (E1s), ubiquitin-conjugating enzymes (E2s), and ubiquitin ligases (E3s), counteracted by deubiquitinases. With 672 curated members (462 classified as “catalytic” (3)) in humans, E3s are the most diverse enzyme class and critical specificity factors in the ubiquitin system (4). They recruit substrates and dictate the nature of ubiquitin modifications, including chains of varied linkage types, topologies and lengths, which determine distinct signaling outcomes.

Based on architecture and catalytic mechanism, E3s are classified into three families: The largest class comprises RING-type E3s, including cullin-RING ligases (5). These enzymes act as scaffolds that juxtapose ubiquitin-loaded E2s with substrates, thereby promoting ubiquitin transfer between them in one step. By contrast, the 28 human homologous to E6AP C-terminus (HECT) and 14 RING-between-RING (RBR)-type ligases catalyze ubiquitin transfer in two steps *via* a thioester-linked E3-ubiquitin intermediate (6, 7). Beyond these canonical E3 classes, atypical ligase mechanisms continue to emerge, highlighting that the range of enzyme architectures driving substrate ubiquitination remains incompletely understood (8, 9, 10, 11, 12, 13, 14).

Owing to their specificity, E3s are prime targets for therapeutic manipulations. In particular, targeted protein degraders, including molecular glues and proteolysis-targeting chimeras, have proven effective in treating hematological malignancies (15). These compounds specifically recruit a disease-causing protein to a degradative E3, promoting proximity-based ubiquitination of the protein and its ensuing proteasomal degradation. While numerous targeted protein degraders are advancing in clinical trials for various indications (16), all of them target RING-type E3s. In contrast, catalytic cysteine-dependent ligases have remained clinically intractable, reflecting our insufficient understanding of their structural mechanisms and ligandability.

This article summarizes recent structural insights into full-length HECT E3s, named after their homology to E6AP (UBE3A), one of the first identified ubiquitin ligases (17). HECT E3s share a C-terminal catalytic cysteine-containing HECT domain that typically allows for a certain degree of autoubiquitination, free ubiquitin chain formation or small molecule ubiquitination (18). Flanking the HECT domain, the ligases feature diverse N-terminal extensions mediating protein substrate recognition, regulation, and localization. Based on the composition of these extensions, two HECT E3 subfamilies have been defined (6): The nine-member human NEDD4 subfamily contains a Ca^2+^-binding C2 domain and multiple WW domains, whereas the six-member HERC (HECT and RCC1-like domain-containing protein) subfamily is characterized by at least one RCC1-like domain (RLD). Notably, HERC5 does not conjugate ubiquitin, but is the principal human E3 for the ubiquitin-like modifier ISG15 (19, 20). The remaining HECT-type ligases have no apparent N-terminal domain commonalities. HECT E3s act across diverse tissues and physiological pathways, and, consequently, their dysfunctions are linked to various human diseases (21). Global phenotypic analyses reveal strong association between HECT E3 variants and neurodevelopmental disorders, indicating critical, but understudied neuronal functions (4, 22, 23).

## Integrating HECT domain mechanism into the full-length E3 context

**Figure 1:**
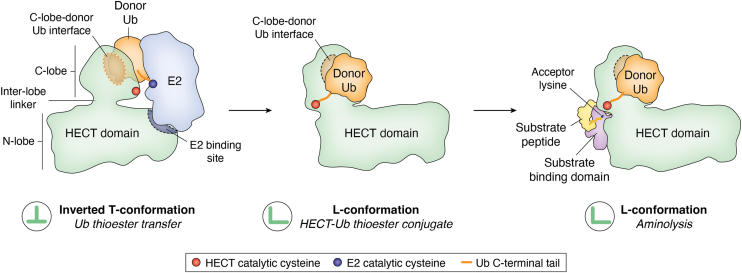
**HECT domain catalysis. Cartoon representation of the conformational cycle of the two-lobe catalytic HECT domain and key reaction steps.***Left*: donor ubiquitin (“Ub”) thioester transfer between the active-site cysteines of E2 and E3, during which the HECT domain adopts an inverted T-conformation; *middle*: transition of the donor Ub-loaded C-lobe of the HECT domain into the L-conformation; *right*: aminolysis, during which an acceptor lysine nucleophilically attacks the C-terminus of the E3-linked donor Ub to give rise to an isopeptide bond between substrate and donor Ub. During this reaction, the HECT domain adopts the L-conformation. The cartoons are based on crystal structures of HECT domain complexes. *Left*: PDB: 3JVZ (NEDD4L HECT domain in complex with a donor Ub-UBE2D2 (E2; *blue*) conjugate) (27); *middle*: PDB: 6XZ1 (HUWE1 HECT domain-donor Ub conjugate) (30); *right*: PDB: 4LCD (WW4 and HECT domain-containing construct of Rsp5 three-way crosslinked to donor Ub and a Sna3-derived substrate peptide (*yellow*)). The peptide is stabilized by the substrate-binding WW4 domain (*purple*). HECT domains are displayed in *green* and donor Ub in *orange*. HECT domain conformations (inverted-T or L) are indicated by *green* symbols in circles. The conserved interface between the donor Ub and the HECT domain C-lobe is indicated by an *orange oval*. The *oval* is encircled by a *black* or *white line*, depending on whether it points toward or away from the viewer. Note that this interface is conserved across the conformational states of the HECT domain. The interface between the E2 and the HECT domain N-lobe is depicted as a *blue half circle*. C-lobe, C-terminal lobe; HECT, homologous to E6AP C-terminus; N-lobe, N-terminal lobe.

Until recently, our structural understanding of HECT E3s centered largely on their hallmark HECT domain. As previously reviewed (6, 24), X-ray crystallographic work converged into a coherent, but incomplete view of the domain's two-step catalytic cycle (Fig. 1): the ∼44-kDa HECT domain consists of a larger N-terminal lobe (N-lobe) and a smaller C-terminal lobe (C-lobe), flexibly tethered by a short linker that facilitates critical inter-lobe movements (25, 26). To transfer a thioester-linked “donor” ubiquitin from an E2 to the active-site cysteine on the HECT C-lobe, the HECT domain adopts an “inverted T”-conformation. In this state, the N-lobe binds the E2, and the C-lobe forms a small, conserved interface with an Ile36-centered hydrophobic patch of ubiquitin that is maintained throughout catalysis (27, 28, 29, 30, 31, 32, 33, 34). Following ubiquitin transfer to the E3, the E2 dissociates and the donor ubiquitin-loaded HECT domain transitions into an “L”-conformation. This allows the C-terminal tail of ubiquitin to be coordinated between the N- and C-lobe, creating a composite active site including the C-terminal extension of the C-lobe (29, 30, 31, 32, 33). A C-terminal carboxyl group of the ligase – provided either by the C-terminus itself or an acidic side chain – contributes to aminolysis (28, 29, 31, 32, 35, 36). Moreover, a ligation-organizing loop of the N-lobe functions in aligning the active site, facilitating the nucleophilic attack of a primary amino group on the activated C-terminal carbonyl of the donor, resulting in aminolysis (29, 31, 32). The amino group may be part of a substrate or an “acceptor" ubiquitin during ubiquitin linkage formation.

Substrate proteins are not recognized by the HECT domain itself, but relie on motifs within the E3's N-terminal regions for recruitment. Likewise, the interactions of isolated HECT domains with acceptor ubiquitin exceed the measurable affinity range, indicating that efficient ubiquitin linkage formation is also promoted by regions outside the HECT domain. Consequently, mechanistic insight into HECT E3-mediated substrate modification and ubiquitin chain assembly requires structural analysis of extended ligase constructs. Aside from their interactions with donor and acceptor ubiquitin, several HECT domains contain a micromolar-affinity ubiquitin binding exosite on the N-lobe that influences ubiquitin chain elongation *via* yet unknown mechanisms (34, 37, 38, 39, 40, 41, 42).

Advances in cryogenic electron microscopy (cryo-EM) have rapidly expanded our structural knowledge of full-length HECT E3s. Over the past 5 years, 50 structures (6 yet to be released) of nine full-length human HECT-type E3s and orthologues have been reported in 20 independent studies, reflecting intense research efforts into this ligase class (Table 1). This body of data provides unprecedented insight into HECT E3 architecture, regulation, and specificity, illustrating how the modular N-terminal scaffolds contribute to ligase functions and highlighting remarkable family-wide diversity. By contrast, the C-terminal HECT domain appears to follow conserved catalytic principles within the full-length protein context, as established in earlier X-ray crystallographic and biochemical work (6, 24).

**Table 1:** Structures of full-length HECT E3 constructs

E3 name	E3 origin	# Residues	# Residues in E3 construct	# E3 residues modelled	% E3 modelled	Oligomeric state	Protein binding partners	Additional description	PDB code
HUWE1	*H. sapiens*	4374	4374	2427	55	Monomer	-	-	7JQ9
	*H. sapiens*	4374	4374	2427	55	Monomer	-	Focussed refinement of HECTdomain	7MWD
	*H. sapiens*	4374	4374	2427	55	Monomer	-	Focussed refinement of WWE domain	7MWE
	*H. sapiens*	4374	4374	2427	55	Monomer	-	Focussed refinement of solenoid interface	7MWF
	*H. sapiens*	4374	4374	2445	56	Monomer	DDIT4	7 residues of DDIT4 substrate modelled	7MOP
HUWE1_N_	*Nematocida sp. ERTm5*	2490	2490	2138	86	Monomer	-	-	7NH1
	*Nematocida sp. ERTm5*	2490	2490	2138	86	Monomer	-	-	7NH3
	*Nematocida sp. ERTm5*	2490	2490	2144	86	Monomer	-	Crystal structure	7BII
Tom1	*S. cerevisiae*	3268	3268	2490	76	Monomer	-		9DNT
	*S. cerevisiae*	3268	3268	2602	80	Monomer	*2 × H. sapiens* UBE2D2, 4 *× H. sapiens* ubiquitin	-	9DNS
	*S. cerevisiae*	3268	3268	2680	82	Monomer	-	His_12_-tag inserted between K1074 and D1075	9ELD
	*S. cerevisiae*	3268	3009	2781	85	Monomer	-	Acidic domain (residues 1873–2131) deleted	9EGK
	*S. cerevisiae*	3268	3268	2683	82	Monomer	-	His_12_-tag inserted between K1074 and D1075	9MHP
UBR5	*H. sapiens*	2799	2799	1654	59	Dimer	-	L710D variant to impair tetramerization	8C06
	*H. sapiens*	2799	2799	503	18	Dimer	2 *×* ubiquitin	L710D variant to impair tetramerization; 3-way crosslink of donor and acceptor Ub to E3 active site; only C-terminal E3 region modelled	8C07
	*H. sapiens*	2799	2799	1644	59	Dimer	-	-	8BJA
	*H. sapiens*	2799	2601	1596	57	Dimer	-	Residues 522–720 deleted to impair tetramerization	8P82
	*H. sapiens*	2799	2799	1759	63	Tetramer	-	-	8P83
	*H. sapiens*	2799	2799	1669	60	Dimer	-	-	8D4X
	*H. sapiens*	2799	2799	1689	60	Dimer	-	-	8E0Q
	*H. sapiens*	2799	2799	1775	63	Tetramer	-	-	8EWI
TRIP12	*H. sapiens*	2040 (isoform 3)	1562	1222	60	Monomer	K29/K48 branched tri-Ub aminolysis intermediate mimic	3-way crosslink of acceptor (K29) and donor Ub to E3 active site; TRIP12 (residues 478–2040)	9GKM
	*H. sapiens*	2040 (isoform 3)	1562	1235	61	Monomer	K29-linked di-Ub aminolysis intermediate mimic	3-way crosslink of acceptor (K29) and donor Ub to E3 active site; TRIP12 (residues 478–2040)	9GKN
	*H. sapiens*	1992 (isoform 1)	1550	1125	56	Monomer	K29/K48 branched *H.sapiens* tri-Ub aminolysis intermediate mimic	3-way crosslink of acceptor (K29) and donor Ub to E3 active site; donor Ub not resolved; TRIP12 (residues 442–1992)	9KEN
Ufd4	*S. cerevisiae*	1483	1483	1234	83	Monomer	K29/K48 branched *H.sapiens* tri-Ub aminolysis intermediate mimic	3-way crosslink of acceptor (K29) and donor Ub to E3 active site	8J1P
	*S. cerevisiae*	1483	1483	677	46	Monomer	*S. cerevisiae* Ubc4-*H. sapiens* Ub thioester transfer mimic	3-way crosslink of donor Ub and E2 active site to E3 active site; Ub not resolved	8J1R
NEDD4L	*H. sapiens*	955 (isoform 5)	955	639	67	Monomer	-	-	9GIK
	*H. sapiens*	955 (isoform 5)	955	639	67	Monomer	-	In the presence of Ca^2+^	9GIM
HACE1	*H. sapiens*	909	909	850	94	Monomer	-	-	8H8X
	*H. sapiens*	909	909	845	93	Dimer	-	-	8HAE
	*H. sapiens*	909	909	830	91	Dimer	-	Only one HECT C-lobe modelled	8PWL
HACE1	*H. sapiens*	909	889	831	91	Monomer	RAC1	Residues 1–20 deleted to impair dimerization; crosslink of RAC1 acceptor site (K147) to E3 active site	8Q0N
E6AP	*H. sapiens*	875	875	681	78	Monomer	*HPV16* E6, P53	-	8R1F
	*H. sapiens*	875	875	681	78	Dimer	*HPV16* E6, P53	-	8R1G
	*H. sapiens*	875	875	692	79	Monomer	*HPV16* E6, P53 (residues 1−312)	-	9CHT
	*H. sapiens*	875	875	689	79	Dimer	*HPV16* E6	-	8JRN
	*H. sapiens*	875	875	689	79	Dimer	*HPV16* E6	-	8JRO
	*H. sapiens*	875	875	689	79	Dimer	*HPV16* E6	-	8JRP
	*H. sapiens*	875	875	676	77	Dimer	*HPV16* E6	-	8JRQ
	*H. sapiens*	875	875	645	74	Dimer	*HPV16* E6	-	8JRR
	*H. sapiens*	875	875	561	64	Monomer	*HPV16* E6, P53 (residues 94–312)	MBP-tagged E6	8GCR
HECTD3	*H. sapiens*	861	864	807	94	Monomer	-	HECT domain in L-conformation	9R8T
	*H. sapiens*	861	864	812	94	Monomer	-	HECT domain in inverted T-conformation	9R94
	*H. sapiens*	861	864	810	94	Monomer	*H. sapiens* Ub	Crosslink of donor Ub to E3 active site	9R85

**Figure 2:**
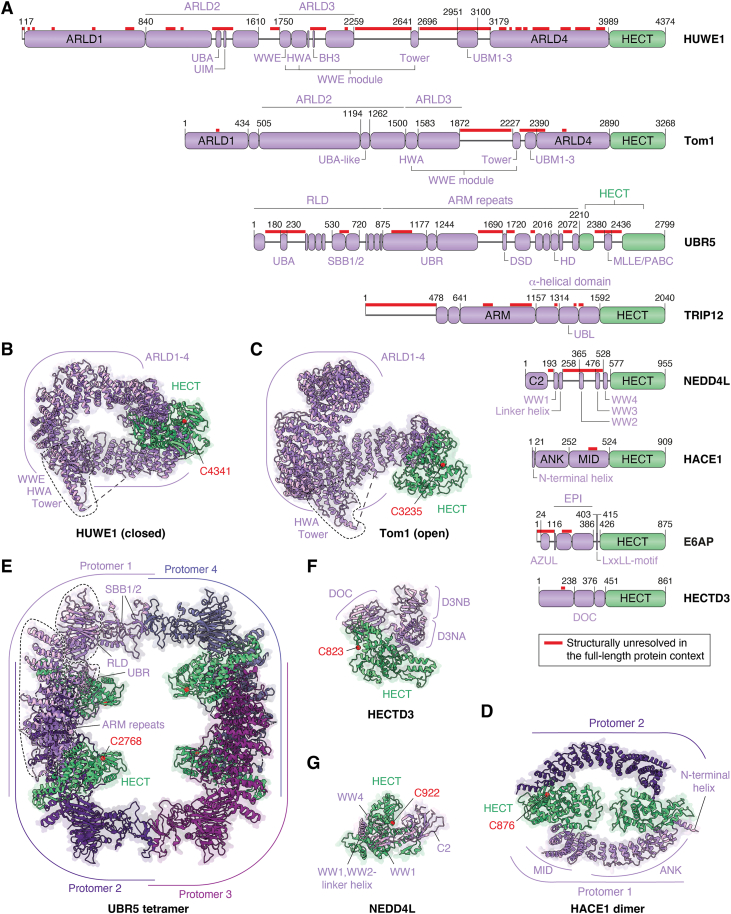
**Full-length HECT E3 architectures.***A,* domain representations of selected HECT E3s of which cryo-EM analyses are featured in this article. For a full list, see Table 1. *Red lines* indicate structurally unresolved regions of >15 residues, based on the following PDBs: HUWE1, 7JQ9 (46); Tom1, 9ELD (45); UBR5, 8EWI (54); TRIP12, 9GKM (32); NEDD4L, 9GIK (69); HACE1, 8PWL (52); E6AP, 9CHT (62); and HECTD3, 9R8T (65). For a list of crystal or nuclear magnetic resonance structures of domains within these unresolved regions, see Table 2. *B,* cryo-EM structure of human HUWE1 (PDB: 7JQ9) (46). *C,* cryo-EM structure of *S. cerevisiae* Tom1 in an open conformation (PDB: 9ELD) (45). *D,* cryo-EM structure of dimeric, human HACE1 (PDB: 8PWL) (52). *E,* cryo-EM structure of tetrameric human UBR5 (PDB: 8EWI) (54). *F,* cryo-EM structure of human HECTD3 (PDB: 9R8T) (65). *G,* cryo-EM structure of human NEDD4L (PDB: 9GIK) (69). In (*A*) to (G), domains are labeled as follows: α-helical domain (TRIP12) = HEL-UBL domain (32); ANK, ankyrin repeat domain; ARLD, armadillo repeat-like domain; ARM, armadillo repeats; AZUL, amino-terminal Zn-finger of UBE3A ligase domain; BH3, BCL-2 Homology three domain; EPI, E6 and P53-interacting domain; HD, HECT display domain; HWA, HUWE1 WWE module-associated domain; LxxLL helix, E6-binding motif (x denotes any amino acid); MID, middle domain; MLLE/PABC, mademoiselle motif/poly(A)-binding protein C-terminal domain; RLD, RRC1-like domain; SBB, small β-barrel; UBA, ubiquitin-associated domain; UBL, ubiquitin-like domain; UBR, UBR box; UIM, ubiquitin-interacting motif; UBM, ubiquitin-binding motif; WW, WW domain; WWE, WWE domain; tower, helix-turn-helix tower domain. In (*D*) and (*E*), domains are labeled only for protomer 1 for clarity. HECT domains are colored *green*, with catalytic cysteines depicted as *red* spheres. Other E3 domains are colored *purple*.

Overview of structures of full-length HECT E3 constructs, released in the PDB. All entries are cryo-EM structures, except PDB: 7BII. The proteins are ordered by size from largest to smallest as in Figure 2*A*. Note that additional unreleased HECT E3 structures have been deposited for UBE3B and UBE3C (PDB: 21RG, 21LW, 21XX, 21LU, 21GC, and 21LV) (64).

# residues, number of amino acids of the respective full-length E3; # residues in E3 construct, number of amino acids in the construct analyzed by cryo-EM; # E3 residues modelled, number of amino acids modelled in the respective structure; in case of oligomers, the count refers to the most structured protomer; Ub, ubiquitin.

## Beyond domains: HECT E3s architectures

Secondary structure and domain predictions originally suggested that the N-terminal regions of HECT E3s may resemble a “beads on a string” arrangement of discrete structured domains interspaced by low-complexity segments (6). Recent structural analyses of full-length ligases (Table 1), together with AlphaFold-based predictions (43), have revised this view, revealing distinct architectural principles. The N-terminal regions of several HECT E3s contain α-helical bundles, giving rise to curved scaffolds of various lengths, flanked by the HECT domain. These scaffolds are decorated with flexible regions that are not visible in current structural models (Fig. 2*A*). Yet, some of these regions contain folded motifs or domains that have been structurally determined in isolation (Table 2).

**Table 2:** Structure of HECT E3 domains/motifs not modelled in the corresponding full-length structures

E3 name	E3 origin	Domain(s)	Construct boundaries	Technique	PDB code
HUWE1	*H. sapiens*	UBA	1317–1356	NMR	2EKK
	*H. sapiens*	UBM1	2951–3003	NMR	2MUL
	*H. sapiens*	BH3-derived peptide (in complex with MCL1)	1969–1994	X-ray crystallography	5C6H
UBR5	*H. sapiens*	MLLE/PABC	2393–2453	X-ray crystallography	1I2T
TRIP12 (isoform 3 numbering)	*H. sapiens*	WWE (ADP-bound)	808–853	X-ray crystallography	9BKS
	*H. sapiens*	WWE (ATP-bound)	807–853	X-ray crystallography	9BKR
NEDD4L (isoform 5 numbering)	*H. sapiens*	WW2 (in complex with phosphorylated SMAD3-derived peptide)	386–400	NMR	2LB2
	*H. sapiens*	WW3 (in complex with phosphorylated SMAD3-derived peptide)	476–515	NMR	2LAJ
	*H. sapiens*	WW2 (in complex with SMAD7-derived peptide)	365–397	NMR	2LTY
	*H. sapiens*	phosphorylated NEDD4L-derived peptide in complex with 14-3-3 γ	338–344	X-ray crystallography	6ZBT
	*H. sapiens*	phosphorylated NEDD4L peptide in complex with 14-3-3 γ	424–430	X-ray crystallography	6ZC9
	*H. sapiens*	WW3	474–512	X-ray crystallography	7LP1
	*H. sapiens*	WW2 (in complex with Angiomotin-derived peptide)	366–398	X-ray crystallography	7LP2
	*H. sapiens*	WW3	473–519	NMR	7LP4
	*H. sapiens*	WW3 (fused with Angiomotin-derived peptide)	473–519	NMR	7LP5
E6AP	*H. sapiens*	AZUL	24–87	NMR	2KR1
	*H. sapiens*	AZUL (in complex with RAZUL domain from hRPN10/S5a)	24–87	NMR	6U19
	*H. sapiens*	AZUL	11–87	NMR	8ENP
	*H. sapiens*	AZUL	1–87	NMR	8EPT

Overview of structures of HECT E3 domains or motifs, located in flexible regions that are marked by *red* lines in Figure 2*A* and could not be modelled in the corresponding full-length HECT E3 structures. The domains are ordered based on the size of the source protein from largest to smallest, as in Figure 2*A*. Note that the residue numbering of constructs provided here was adapted to match the isoforms shown in Figure 2*A*.

Illustrating this theme, cryo-EM structures of human HUWE1 (482 kDa) – consistent with analyses of more compact homologs from *Nematocida sp. ERTm5* (HUWE1_N_; 287 kDa) (44) and *Saccharomyces cerevisiae* (Tom1; 374 kDa) (42, 45) – reveal extended armadillo-like repeat domains (ARLDs), surrounded by structurally unresolved insertions and a module of poly-ADP ribose-binding WWE and HWA (HUWE1 WWE module-associated) (46). The α-helical scaffold assembles into a solenoid of ∼140 × 100 Å from which the HECT domain and a tower domain protrude at opposite sides (Fig. 2*B*). Aside from the closed solenoid state, open and extended conformations have been observed in HUWE1, HUWE1_N_, and Tom1, indicating high plasticity of the α-helical scaffold (Fig. 2*C*) (42, 44, 45, 46). While the intramolecular interface underlying the closed state was found to be required for activity, the functional significance of the open conformations remains to be assessed. Notably, available cryo-EM models account for just 55% of the full-length HUWE1 sequence. The unresolved insertions embed functionally important elements, such as a MCL1-interacting BH3 domain (47) and ubiquitin-binding modules (UBA, UIM, and UBM), indicating allosteric communication of disparate ligase regions with the HECT domain. The conformational pliability of the insertions may allow HUWE1 to accommodate both specific substrates, like MCL1 (47), and a broad range of quality-control targets, including unassembled (“orphan”) subunits of protein complexes (44, 48, 49). Moreover, it may facilitate dynamic rearrangements during ubiquitin chain elongation, a mechanistic specialization of certain HECT E3s, such as HUWE1 and UBR5 (50, 51).

A ring-like architecture of similar size to the folded portion of HUWE1 is observed in a dimeric, autoinhibited state of human HACE1 (102 kDa). Here, the α-helical scaffold is made up of ankyrin repeats, and dimer dissociation is required for catalytic activity (Fig. 2*D*) (52, 53). In contrast, human UBR5 (309 kDa) assembles into a tetramer of U-shaped dimeric constituents, resulting in a larger ring-like structure of ∼230 × 220 Å (Fig. 2*E*) (31, 51, 54, 55, 56). Its α-helical core contains armadillo as well as HEAT repeats and integrates the UBR-box proposed to recognize substrate N-termini (57, 58, 59). The N-terminal region of UBR5 comprises an RLD, interspersed with tandem SBB domains required for tetramerization, and a structurally unresolved UBA domain. Moreover, a substrate-recruiting MLLE/PABC domain is inserted into the HECT domain, but not visible in current cryo-EM maps. The HECT domain interacts with two extended elements, termed domain swap dimerization and HECT display (also known as “plug-loop” (51)) domains, which emanate from the extended α-helical UBR5 scaffold.

As discussed below, a more compact α-helical-repeat scaffold is seen in E6AP (60, 61, 62, 63), as well as UBE3B and UBE3C (64). In contrast, the N-terminal region of HECTD3 lacks α-helical repeats and folds into a unique α-helical domain (D3NA), into which a β-strand-rich unit (D3NB) and a flexible, substrate-binding DOC domain are inserted (Fig. 2*F*) (65). Despite being structurally mostly resolved, the organization of HECTD3 may allow for considerable inter-domain plasticity. Likewise, members of the NEDD4 subfamily lack α-helical bundles and instead adopt dynamic assemblies of flexibly linked domains. Consistent with previous X-ray crystallographic analyses (66, 67, 68), this organizational principle is illustrated by cryo-EM structures of full-length NEDD4L, resolving the membrane-targeting C2 domain, two of the four WW domains, and the HECT domain in an autoinhibited conformation, with the majority of inter-domain linkers remaining unresolved (Fig. 2*G*) (69).

## Snapshots of HECT E3 catalysis: how N-terminal domains guide ubiquitin linkage choice

How a full-length HECT E3 accepts donor ubiquitin from an E2 and encodes linkage specificity during aminolysis was revealed for UBR5 (31). The studies employed a mutated UBR5 variant, that preserves activity but disrupts tetramerization, and tailored three-way crosslinking to mimic reaction intermediates of the ligase dimer. A cryo-EM map for a complex of UBR5 with E2 and donor ubiquitin is consistent with thioester transfer as previously defined in the HECT domain context (29), showing the HECT domain in an inverted T-conformation with donor ubiquitin and E2 oriented in a canonical manner. In this state, the donor forms an interface with the HECT C-lobe that is conserved in the consecutive reaction step.

**Figure 3:**
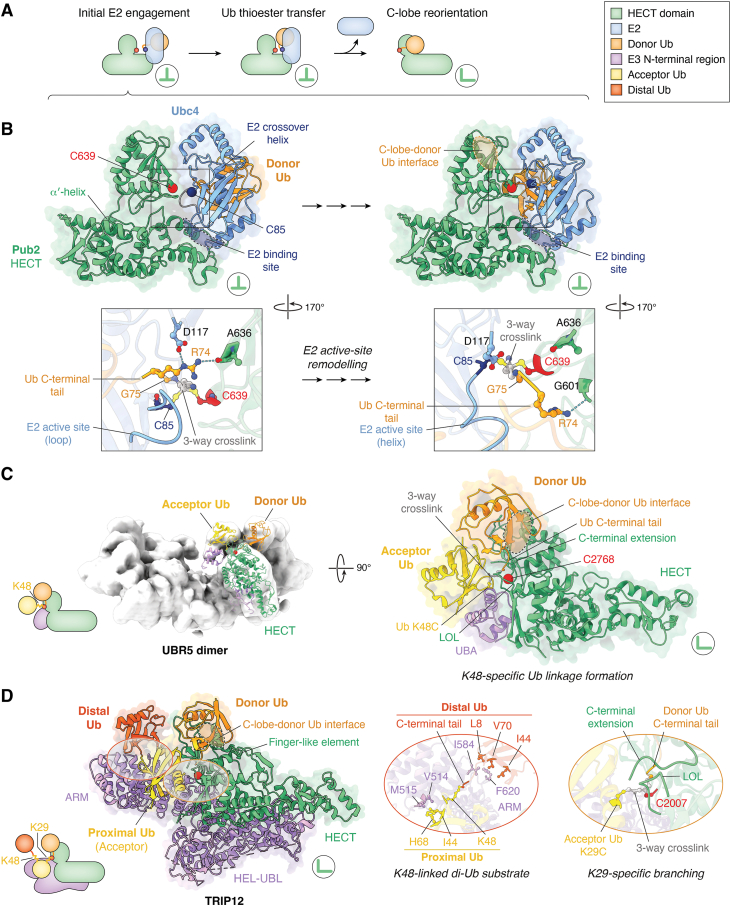
**Structural mechanisms of ubiquitin linkage formation.***A, cartoon* showing distinct E2-Ub-HECT domain configurations formed prior to and during Ub thioester transfer, providing context for the structures in (*B*). Initially, the E2-bound donor Ub engages the E2 crossover helix and the E2 associates with the HECT domain N-lobe (*left*). Thereafter, dynamic active-site remodeling gradually positions the donor toward the HECT domain C-lobe for thioester transfer (*middle*). This reaction is followed by E2 release and rotation of the C-lobe into the L-conformation (*right*). *B, top*: selected cryo-EM structures of the HECT domain of Pub2 three-way crosslinked to the E2 Ubc4 (*blue*) and donor Ub (PDBs: 9B55, and 9B5B; all proteins from *S. pombe*) (70), approximating different stages of Ub thioester transfer between E2 and E3. The *triple arrow* indicates that additional structures presumably representing intermediates between the selected states are available (70). The active-site cysteine Cα-atoms of E2 and E3 are shown as *blue* and *red spheres*, respectively. Note that the C-lobe-donor Ub interface, encircled by a *white dashed line* indicating that it points away from the viewer, is not formed, yet. Instead, the donor Ub contacts the E2 crossover helix (labeled). *Bottom*: detailed view, highlighting remodeling of the E2 active site residues 86 to 92 from loop to helix conformation and key contacting residues at the active site. C639, Pub2 catalytic cysteine; C85, Ubc4 catalytic cysteine. The three-way crosslink between the two catalytic cysteines and the ubiquitin C-terminal tail is based on PSAN (bis-electrophilic 3-[phenylsulfonyl]-4-aminobut-2-enenitrile). The crosslink and selected residues are colored by atom. *C, left*: cartoon of the reconstituted UBR5 complex with donor and acceptor Ub shown in this figure. Note that the acceptor ubiquitin is crosslinked to the E3 active site *via* residue 48, recapitulating Lys48-specific linkage formation. *Middle*: cryo-EM map (EMDB: 17466) of a symmetric UBR5 dimer covalently linked to donor and acceptor Ub, approximating the aminolysis intermediate during Lys48 linkage formation; the corresponding structure of the HECT domain with donor and acceptor, resulting from a focused refinement (PDB: 8C07) (31), is fitted into the map. *Right*: Detailed view of the structure with key elements labelled. C2768, UBR5 catalytic cysteine. The three-way crosslink between the E3 active site cysteine, the acceptor residue 48 (lysine replaced by cysteine; K48C) and the C-terminal tail of the donor Ub is based on BmDPA ((E)-3-[2-(bromomethyl)-1,3-dioxolan-2-yl]prop-2-en-1-amine). *D, left*: cartoon of the reconstituted TRIP12 complex with donor Ub and a Lys48-linked di-Ub chain composed of a proximal acceptor Ub and a distal Ub moiety. Note that the acceptor is crosslinked to the E3 active site *via* residue 29, recapitulating Lys29-specific Ub chain branching. *Middle*: cryo-EM structure of TRIP12 covalently linked to donor and acceptor ubiquitin, with the acceptor being the proximal member of a Lys48-linked ubiquitin chain, approximating the aminolysis intermediate during formation of a Lys29-linked branch (PDB: 9GKM) (32). *Right*: detailed views of key elements and residues. Hydrophobic side chain contacts of the proximal and distal ubiquitin, respectively, with the ARM domain are featured (*middle*). The three-way crosslink is based on BmDPA, as in (C); in this case, the acceptor Lys29 was replaced by cysteine (K29C). C2007, TRIP12 catalytic cysteine. Across all panels, HECT domains are colored *green*, other E3 domains *purple*, donor Ub *orange*, and acceptor Ub *yellow*; the E3 active-site cysteine Cα-atom is shown as a *red sphere*. HECT domain conformations (inverted-T or L) are indicated by *green* symbols in circles. A color code for all the cartoons is provided in the *top right corner*. LOL, ligation-organizing loop; UBA, ubiquitin-associated domain; ARM, armadillo repeats; HEL-UBL domain, Helical scaffold and ubiquitin-like domain.

Cryo-EM studies of ubiquitin transfer between the E2 Ubc4 and the HECT domain of Pub2 from *S. pombe* uncovered a series of states presumably preceding this intermediate (Fig. 3, *A* and *B*) (70). Here, the donor ubiquitin, three-way crosslinked to E2 and E3 approximating the thioester transfer intermediate in a near-native fashion, interacts with the E2 crossover helix (Fig. 3*B*), and the C-terminal ubiquitin tail displaces residues near the E2 active site. These residues form a loop that interacts with the HECT N-lobe and positions the ubiquitin C-terminal tail (*via* Arg74) to contact both the E2 and the HECT C-lobe. Movement of ubiquitin from the E2 toward the HECT C-lobe is accompanied by remodeling of the loop into a helical conformation, likely facilitating ubiquitin release from the E2. This remodeling may allow the ligase to discriminate ubiquitin-loaded from free E2, thereby preventing reversibility of the isoenergetic thioester transfer, in-line with biochemical observations (35, 71, 72).

Following ubiquitin transfer to the E3, the C-lobe transitions into the L-conformation, while maintaining its interface with the donor (Fig. 3*A*). This state was characterized for full-length UBR5 and HECTD3 by cryo-EM (31, 65), in line with previous X-ray crystallographic work (29, 30). Structural analyses of full-length UBR5 further demonstrate that the transition involves coordinated rearrangements of the HECT domain relative to the α-helical scaffold, occluding the E2-binding surface on the N-lobe (31). These rearrangements point to a steric gating mechanism, further enforcing catalytic directionality.

To define the structural basis of linkage specificity during aminolysis, a three-way crosslinked complex of UBR5 with donor and acceptor ubiquitin was reconstituted (Fig. 3*C*) (31). The acceptor was installed with Lys48 at the E3 active site, consistent with the intrinsic linkage preference of UBR5 (73, 74). The resulting structure shows the HECT domain in the L-conformation, with donor ubiquitin in the conserved orientation. The acceptor ubiquitin – visualized here for the first time in a HECT E3 complex – is coordinated by the donor ubiquitin, the β-sheet of the HECT C-lobe and the UBA domain of UBR5, illustrating how N-terminal ligase domains contribute to linkage specificity. The composite active site comprises the catalytic cysteine-bearing loop, the C-terminal extension of the C-lobe, the ligation-organizing loop of the N-lobe, and the C-terminal tail of the donor ubiquitin, in line with previous models (29, 30, 33).

Notably, all macromolecular complexes captured for dimeric UBR5 are compatible with the tetrameric state. Higher-order assembly of the ligase may enable subunit cooperation during substrate modification and ubiquitin chain elongation, for example by allowing the flexibly tethered UBA domain to stabilize the acceptor ubiquitin across subunits *in trans*. Such cooperation, together with avidity and kinetic effects (allovalency) on substrate or ubiquitin binding, may underlie the processivity of UBR5-driven ubiquitin chain formation (31, 51, 56).

Several HECT E3s, including UBR5, HUWE1, and TRIP12 can catalyze ubiquitin chain branching (32, 73, 74, 75, 76, 77, 78). The structural basis of this capacity was elucidated for human TRIP12 (32, 78) and its *S. cerevisiae* homolog Ufd4 (78). Guided by biochemical data demonstrating Lys29-specific linkage formation on the proximal ubiquitin unit within a Lys48-linked di-ubiquitin chain, a three-way crosslinked complex of TRIP12, donor ubiquitin, and a di-ubiquitin acceptor was assembled (Fig. 3*D*) (32). Here, an intrinsically disordered N-terminal region of the E3 was truncated to enable structure determination. The ligase architecture comprises armadillo repeats, followed by a central α-helical domain with a UBL domain insertion (referred to as “HEL-UBL” (32)), and the C-terminal HECT domain. The armadillo repeats interact with the Ile44-centered hydrophobic patches of the di-ubiquitin substrate, orienting the proximal acceptor moiety toward the catalytic center. Notably, the arrangement of the tandem Ub binding sites in TRIP12 is only compatible with a Lys48-linked chain, explaining the substrate preference of the ligase during branch formation and illustrating how the N-terminal ligase domains contribute to it. In the ternary complex, the HECT domain adopts an L-conformation, with donor ubiquitin stabilized in the conserved orientation by an atypical finger-like element of the N-lobe. Together, this architecture allows the acceptor ubiquitin to nestle into a complementary surface of the composite active site, organized analogously to UBR5 (31); in this case, however, Lys29 is juxtaposed with the C-terminus of the donor. The same structural arrangement is observed with mono-ubiquitin rather than di-ubiquitin as an acceptor, reflecting the inherent linkage specificity of TRIP12, independent of its branching activity (32).

Analogous complexes of TRIP12 and Ufd4 assembled by alternative three-way crosslinking strategies show overall similar acceptor positioning (78). In Ufd4, however, the donor ubiquitin adopts an atypical orientation relative to the HECT C-lobe, the origin and functional relevance of which remain unclear.

## Shared autoinhibition, distinct structural solutions

Many HECT E3s adopt autoinhibited conformations, reflecting a physiological need for stringent control of their activity (6, 24). Consistent with their diverse N-terminal architectures, inhibitory mechanisms in HECT E3s are enzyme-specific and comprise intramolecular interactions (66, 67, 68, 69, 79, 80, 81, 82, 83, 84), oligomerization, (52, 53, 64, 85, 86), and interactions with macromolecular partners (69, 87, 88). Likewise, diverse activating mechanisms have been described, including phosphorylation, (52, 67, 68, 84, 89, 90, 91, 92, 93), oligomerization (94), Ca^2+^ or lipid binding (64, 69, 80, 95, 96, 97), and engagement of substrates or regulating factors (68, 87, 97, 98, 99, 100, 101). Despite this diversity, a common regulatory principle emerges: autoinhibition of HECT E3s typically impairs the first catalytic step. This is analogous to autoinhibition modes described in E2s and RBR-type E3s, which also form thioester-linked intermediates with ubiquitin. For example, thioester transfer between E1 and E2 can be regulated by E2 dimerization, autoubiquitination, phosphorylation or interactions with lipids (102, 103, 104, 105). Thioester transfer from E2 to RBR-type ligases is, in turn, modulated by E3-specific autoinhibitory domain interactions that modulate the accessibility of the E2 binding site or of the catalytic cysteine (7, 106).

**Figure 4:**
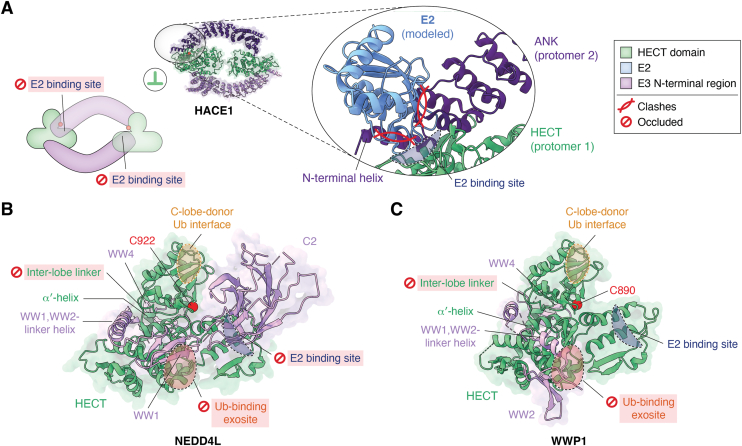
**Autoinhibition modes of full-length HECT E3s.***A, bottom left*: cartoon showing the autoinhibited, symmetric HACE1 dimer, in which the E2 binding sites on both protomers are occluded at the dimer interface. *Topleft*: cryo-EM structure of the HACE1 dimer (PDB: 8PWL) (52) and detailed view (*middle*), with an E2 (UBE2D2) modelled in the conserved orientation required for Ub thioester transfer (based on PDB: 3JVZ (27)). Steric clashes between the E2 and the HACE1 ankyrin repeats (ANK) at the dimer interface are indicated by *red symbols*. *Right*: Color and symbol legend for (*A*)–(*C*). *B,* cryo-EM structure of autoinhibited NEDD4L, highlighting autoinhibitory features and interfaces (PDB: 9GIK) (69). The WW2 and WW3 domains and several linkers are not resolved. The Ub-binding exosite of the HECT domain N-lobe (*red oval*) is encircled by a *black dashed line*, as it points toward the viewer. Likewise, the E2 binding site of the HECT domain N-lobe, depicted by a *blue half circle*, points toward the viewer. In contrast, the C-lobe-donor Ub interface (*orange oval*) is encircled by a *white dashed line*, indicating that it points away from the viewer. Note that the inter-lobe linker, the E2 binding site, and the ubiquitin-binding exosite are occluded by autoinhibitory domain interactions, while the donor Ub binding site is accessible. *C,* crystal structure of a WWP1 construct starting with the WW2 domain, highlighting autoinhibitory features and interfaces (PDB: 6J1X) (66), in line with earlier work (67, 68). Interfaces are depicted as in (*B*). The WW3 domain is not resolved. Note that the exosite and the inter-lobe linker are occluded by autoinhibitory domain interactions, while the donor Ub and E2 binding sites are accessible. The HECT domains in (*B*) and (*C*) are shown in the same orientation for comparison, with Cα-atoms of catalytic cysteines shown as *red spheres*. C890, WWP1 catalytic cysteine; WW, WW domain; N-lobe, N-terminal lobe; C922, NEDD4L catalytic cysteine.

How autoinhibition can be structurally implemented in a full-length HECT E3 was illustrated for HACE1, a tumor suppressor and neurodevelopmental regulator (107). HACE1 forms a symmetric dimer whose subunit interface occludes the E2 binding site on the HECT N-lobe (Figs. 2*D* and 4*A*) (52, 53). Dimerization therefore inhibits ubiquitin thioester transfer from E2 to E3, even though the HECT C-lobe remains flexible and the active-site cysteine accessible. Several physiological phosphorylation events in HACE1 map to the hydrophobic dimer interface and are incompatible with dimerization, suggesting that phosphorylation stabilizes the active monomeric state (52). The upstream triggers and kinases governing this conformational transition, however, remain unknown.

NEDD4-subfamily ligases are regulated by complex inter-domain dynamics, as reviewed elsewhere (24, 108). In brief, nuclear magnetic resonance analyses of SMURF2 revealed that its N-terminal C2 domain mediates autoinhibition by preventing ubiquitin thioester transfer to the C-lobe and antagonizing ubiquitin binding to the regulatory exosite (79, 80, 81). The recent cryo-EM structure of full-length NEDD4L shows the C2 domain obstructing the E2 binding site, in-line with autoinhibition affecting the first catalytic step. Additional inhibitory domain interfaces seen in this structure are consistent with previous X-ray crystallographic work on WWP1, WWP2, and ITCH constructs lacking the C2 domain (66, 67, 68). Those studies revealed how the WW domains obstruct functionally important elements of the HECT domain, such as the ubiquitin-binding exosite (Fig. 4, *B* and *C*). An α-helical inter-WW domain linker reinforces this inhibition by occluding the HECT domain inter-lobe linker, constraining the C-lobe in an unproductive state and preventing ubiquitin thioester transfer. Diverse mechanisms can relieve this multilayered autoinhibition, including phosphorylation of the inhibitory inter-WW domain linker, binding of the WW domains to substrates or adaptors, and conformational rearrangements imposed by interactions of the C2 domain with Ca^2+^ and membrane lipids (66, 67, 68, 69, 82, 87, 95, 96).

The regulatory mechanisms of other HECT E3s for which full-length structures are available remain less well defined. For example, it is unknown whether opening of the solenoid scaffold in HUWE1/Tom1 serves a regulatory purpose or whether distinct oligomeric states of UBR5 and E6AP differentially control substrate ubiquitination.

## Substrate recognition in HECT ligases: customization as the guiding principle

**Figure 5:**
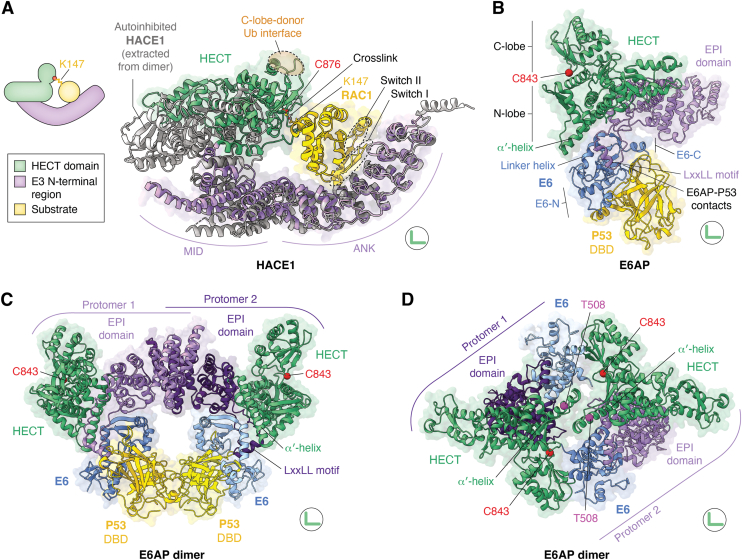
**Structural modes of substrate recognition by HECT E3s.***A, left*: *cartoon* of the reconstituted HACE1-RAC1 complex shown in the structure. K147, the major HACE1-modifed site of RAC1 is crosslinked to the E3 catalytic cysteine. A *color code* is provided below. *Right*: cryo-EM structure of an active monomeric HACE1 version that lacks 20 N-terminal residues required for dimerization, crosslinked to constitutively active, GTP-loaded RAC1 Q61L (PDB: 8Q0N; shown in *green, purple* and *yellow*) (52). The crosslink between the E3 active site and the RAC1 ubiquitination site is based on SIA (N-succinimidyl iodoacetate); C876, catalytic cysteine of HACE1. The structure is superposed *via* the ankyrin repeats (ANK) with that of an inactive full-length HACE1 protomer (containing the N-terminal α-helix), extracted from the autoinhibited dimer (*gray*; PDB: 8PWL), visualizing the conformational changes in the MID and HECT domain upon RAC1 binding (52). Note that the RAC1-bound conformation is compatible with the engagement of a donor ubiquitin at the conserved C-lobe site (*orange oval*). *B,* cryo-EM structure of monomeric E6AP in complex with *HPV* E6 (*blue*) and the P53 DBD (*yellow*; PDB: 9CHT) (62). *C,* cryo-EM structure of dimeric E6AP in complex with *HPV* E6 and P53, captured by glutaraldehyde crosslinking (PDB: 8R1G) (60). *D,* cryo-EM structure of dimeric E6AP in complex with *HPV* E6 (PDB: 8JRN) (61). The Cα-atom of T508, a disease-associated phosphorylation site within the α′-helix of E6AP is highlighted in *magenta*. HECT domains are colored *green* and other E3 domains *purple*; the Cα-atoms of E3 catalytic cysteines displayed as *red spheres*. C843, E6AP catalytic cysteine; EPI, E6 and P53-interacting domain; E6-N, E6 N-terminal domain; E6-C, E6 C-terminal domain; linker helix, E6-N and E6-C linker; DBD, DNA-binding domain. LxxLL helix, E6-binding motif (x denotes any amino acid).

How HECT E3s position substrates for ubiquitination has been difficult to resolve structurally, owing to the transient and weak nature of these interactions. To overcome this limitation, cryo-EM studies of a monomeric, active HACE1 in complex with its major physiological substrate, the small GTPase RAC1 (109), employed a mechanism-based crosslinking strategy (110), covalently tethering the catalytic cysteine of HACE1 to the principal ubiquitination site of RAC1 (52). In the resulting complex, RAC1 binds the concave face of the ankyrin-repeat scaffold, and the HECT domain is markedly rearranged relative to the dimeric autoinhibited state (Fig. 5*A*). The catalytic C-lobe adopts the L-conformation required for aminolysis and could, in principle, interact with a donor ubiquitin in a canonical orientation. Notably, the RAC1 binding mode rationalizes HACE1 specificity for active, GTP-loaded over inactive, GDP-bound RAC1, as the nucleotide-sensitive switch regions of the GTPase fold are stabilized at the ligase interface. HACE1 thus not only confers substrate selectivity but discriminates between conformational states of a given substrate to determine signaling specificity. Beyond RAC1 and a few other substrates, the target spectrum of HACE1 remains poorly defined. It will thus be important to investigate whether the rather compact architecture of this ligase accommodates diverse substrate folds – possibly *via* the flexible insertion within its α-helical middle domain (Fig. 2*A*) – or whether HACE1 modifies a specialized substrate range, including select small GTPases (109, 111).

A different mechanism of substrate recruitment is seen in E6AP. To promote ubiquitination and proteasomal degradation of the P53 tumor suppressor, this E3 is hijacked by the human papillomavirus (HPV) E6 protein that functions as a substrate adaptor (112). While X-ray crystallographic studies revealed how an E6AP-derived LxxLL-peptide recruits both components (113, 114, 115), recent cryo-EM work provided the first structural views of full-length E6AP complexes with E6 and P53 (60, 62, 63). The ligase adopts a compact fold, with an α-helix-rich E6 and P53-interacting (EPI) domain adjacent to the HECT N-lobe. The LxxLL-motif is a flexibly tethered insertion within the EPI scaffold and binds a pocket formed by two E6 parts (E6-N and E6-C) and a linker helix between them (60, 61, 62, 63, 114). The E6 protein is further sandwiched between the EPI domain and the α′-helix of the E6AP N-lobe, resulting in a large (∼2300 Å^2^) interface of picomolar affinity (Fig. 5*B*) (63). P53 binding to E6 is mediated by both the E6-N and E6-C domains. In addition to E6AP stabilizing a P53 binding-competent conformation of E6, several contacts between a loop preceding the E6AP LxxLL-motif and P53 are observed in some of the structures and contribute to efficient P53 ubiquitination.

Aside from ternary assemblies containing monomeric E6AP, a higher-order complex including dimeric E6AP was trapped by glutaraldehyde crosslinking (Fig. 5*C*) (60). In this complex, the E6AP protomers interact *via* their EPI domains, while the orientation of E6 and P53 toward each ligase protomer is maintained compared to the above-described ternary arrangement. Notably, in both assemblies, P53 is removed from the catalytic center of the HECT C-lobe, suggesting that structural rearrangements or other oligomerization modes are required to confer P53 ubiquitination.

Additional cryo-EM studies of an E6-E6AP complex in the absence of P53 reveal a distinct head-to-tail mode of E6AP dimerization, mediated by inter-subunit contacts between the N-lobe α′-helices (Fig. 5*D*) (61). Here, the HECT C-lobe of one E6AP protomer is positioned near an E6 molecule bound to the other ligase protomer. Considering C-lobe flexibility, the arrangement is compatible with the binding mode of P53 seen in the ternary complexes and may allow P53 ubiquitination *in trans*. However, further analyses are required to test this idea and ultimately define the E6AP conformation required for P53 ubiquitination.

Together, these studies highlight the significance of the N-lobe α′-helix for both substrate binding and oligomerization of E6AP (60, 61, 62, 63, 116). Conformational malleability of the C-terminal part of this helix, as seen across different cryo-EM maps and MD simulations, may be involved in ligase regulation (61). Consistent with this notion, several loss-of-function Angelman-syndrome mutations coincide with this region, disrupting its α-helical structure (*e.g.*, R500P and R505P) (61, 90, 117, 118). The significance of this region for E6AP activity is also reflected by a regulatory phosphorylation site, Thr508, that is altered by a gain-of-function mutation (T508A) associated with autism-spectrum disorders (Fig. 5*D*) (90). Notably, beyond E6AP, the α′-helix has emerged as a determinant of HECT domain stability and function that is modulated by posttranslational modifications in several HECT E3s (86, 119, 120).

Beyond the substrate-specific binding modes described for HACE1 and E6AP, how broadly acting quality-control ligases recognize diverse substrates, including orphan subunits and misfolded proteins, remains unclear. In recent structural work on UBR5, two substrate-binding modes have been proposed: Low-resolution negative-stain EM of a dimeric UBR5 construct lacking the tandem SBB and HECT domains implicated the RLD, armadillo-repeat scaffold, and UBR domains in binding to a retinoic acid receptor-α (RARA)–RXRA heterodimer. By contrast, integrated modeling of another substrate, the INO80 remodeling complex subunit MCRS1, into a low-resolution cryo-EM map of tetrameric UBR5 positioned the degron-containing FHA domain of MCRS1 between the HECT C-lobe of one protomer and the SBB domains of another (56). Whether this arrangement represents a productive ubiquitination complex remains to determined, as the modeled FHA domain would conflict with a canonical positioning of the donor ubiquitin. In the same vein, further analyses are required to understand the division of labor among UBR5 protomers during substrate ubiquitination.

In HUWE1, substrate recognition can occur *via* dedicated receptors, such as the MCL1-binding BH3 domain (47), or a disordered acidic loop that recruits unassembled histones (44, 45). In addition, the ARLD scaffold is thought to bind unfolded or extended substrate regions through its concave surface, based on electron density originating from a DDIT4 (DNA damage-inducible transcript 4 protein) peptide in cryo-EM maps of the substrate-bound ligase (46). Analogous to HACE1, this suggests that the α-helical scaffold of HUWE1 provides a versatile substrate-binding platform, consistent with established roles of ankyrin and armadillo repeats in protein–protein interactions (121). Notably, modification of diverse substrates within the HUWE1 solenoid would require substantial HECT-domain flexibility. Although current reconstructions capture the HECT domain predominantly in inverted T-like conformations sterically incompatible with the L-state, cryo-EM and crosslinking analyses support such rearrangements, enabled by a hinge adjacent to the HECT domain (44, 46).

## Toward understanding higher-order HECT E3 assemblies

**Figure 6:**
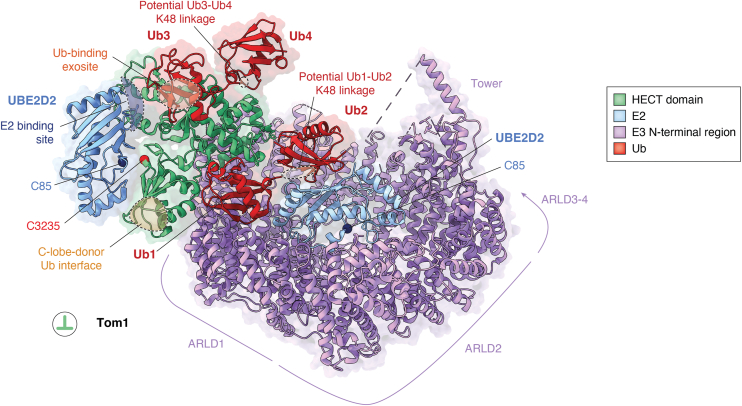
**Higher-order reaction-derived Tom1 complex.** Cryo-EM structure of a complex of Tom1 with four ubiquitin (*red*) and 2 UBE2D2 (E2; *blue*) molecules (PDB: 9DNS) (42). C85, UBE2D2 catalytic cysteine; C3235, Tom1 catalytic cysteine. Potential Lys48 linkages between ubiquitin molecules as indicated. The Tom1 HECT domain is colored *green*, with the catalytic cysteine Cα-atom shown as a *red sphere*. The remainder of Tom1 is colored *purple*. UBE2D2 catalytic cysteine positions are depicted with *dark blue spheres*.

Aside from targeted crosslinking approaches, structural snapshots of HECT E3 complexes were obtained by rapid vitrification of Tom1 and UBE3C-driven ubiquitination reactions, respectively (42, 101). As the corresponding UBE3C structures, characterized in the context of the 28S proteasome, have not been released, yet, we limit the following discussion to Tom1. Here, reaction mixtures contained E1, ATP/Mg^2+^, ubiquitin, E2 (UBE2D2), and histone H2B, a physiological Tom1/HUWE1 substrate (122, 123). Heterogeneous refinement resolved closed solenoid and open states of the ligase, as well as complexes of the closed form with E2 and ubiquitin molecules. All of these complexes show the HECT domain in the inverted T-conformation and a ubiquitin molecule (Ub1) coordinated by the HECT domain and the ARLD1 (Fig. 6). Among the different structures, Ub1 is either a mono-ubiquitin or the distal member of a Lys48-linked di-ubiquitin with Ub2 (linkage not modelled). A mutation (L615R) at the ARLD1–Ub1 interface specifically reduces Lys48-linked ubiquitin chain formation by Tom1, implicating ARLD1 in linkage selectivity.

While UBE2D2 occupies the canonical E2-binding site on the HECT N-lobe, an unexpected second binding mode is observed in some of the structures, positioning the E2 between ARLD1, ARLD2, and the Ub1-Ub2 chain. Neither E2 carries a donor ubiquitin, nor is donor ubiquitin detected on the Tom1 active site. Ub2 engages the E2 backside, reminiscent of RING E3 catalysis (124, 125). The relevance of this atypical E2 engagement, however, remains unclear.

Two further ubiquitin molecules (Ub3, Ub4) associate with the HECT N-lobe of Tom1. Their orientation is compatible with a Lys48 linkage, with the distal ubiquitin unit (Ub3) occupying the conserved exosite. This supports the notion that the exosite promotes chain elongation by engaging a growing ubiquitin chain (6, 24, 101). Yet, all of the Tom1 complexes show a HECT domain position incompatible with the L-conformation required for aminolysis (30). Structural rearrangements as seen in *apo* Tom1 structures could permit the HECT domain to adopt an L-conformation, suggesting that yet uncharacterized conformational transitions accompany catalysis. It will also be important to define the contributions of the structurally unresolved ubiquitin-binding modules of Tom1, which may further expand the complexity of higher-order catalytic assemblies.

## Structures in hand, new questions in abundance

Structural studies of full-length HECT E3s have revealed remarkable diversity in molecular organization, oligomerization, regulation, and specificity across the ligase family, consistent with the heterogeneity of the enzymes' N-terminal regions. In contrast, the C-terminal HECT domain follows conserved catalytic principles that are shared between the currently characterized set of full-length enzymes and align with previous insights from isolated HECT domains (6). Together, these advances highlight key questions for future structural work.

### Navigating the increasingly complex landscape of HECT E3 complexes

The complicated higher-order configurations observed in reaction–derived assemblies of Tom1 (42) and UBE3C (101) underscore the need to functionally distinguish individual interfaces of HECT E3s with distinct ubiquitin molecules, ubiquitin chains, and reaction partners. Such discrimination will be essential to differentiate productive, on-pathway intermediates from autoinhibited or non-native complexes. Time-resolved structural approaches, together with advanced biochemical kinetics, may help address this challenge, enabling discrete configurations to be ordered along reaction coordinates. In parallel, substrate dependencies of catalytic E3 assemblies and associated processivity require systematic investigation.

### Understanding ubiquitin chain formation on substrates

Structural studies of HACE1 elucidated the molecular basis of ubiquitin transfer to a defined substrate lysine (52), while analyses of UBR5 and TRIP12/Ufd4 revealed how linkage specificity is encoded in the absence of a substrate (31, 32, 78). A major outstanding challenge is to define how HECT E3s assemble ubiquitin chains on substrates. Chain elongation likely requires coordinated rearrangements of the HECT domain, the substrate, and the growing ubiquitin chain, which may impose as-yet-uncharacterized constraints on chain length and topology. In this context, it will also be important to define the roles of accessory ubiquitin binding sites for processivity in chain formation, chain length regulation, linkage specificity, and branching.

### Defining substrate recognition principles across HECT E3s

Beyond the NEDD4 subfamily, in which WW domains mediate recognition of PPxY motifs (126), and few other identified ligase-specific substrate binding modes, the substrate specificities of many HECT E3 remain incompletely defined. Large quality-control enzymes, such as HUWE1 and UBR5, can recruit substrates either through discrete motifs or broader surface features, whereas some smaller HECT E3s, act on folded substrates of diverse architectures. Deriving principles of substrate recognition will thus require structural characterization of diverse HECT E3–substrate complexes, together with clarification of the functional contributions of flexible ligase regions that have remained structurally unresolved. In addition, the spatiotemporal availability of substrates to a given E3 – a frequently unexplored dimension (22) – requires systematic investigation in cellular contexts.

### Elucidating HECT E3 roles in atypical ubiquitination

The discovery of oxyester-linked ubiquitin on proteins and non-protein biomolecules necessitates a reassessment of E3 specificities (8, 10). An important open question is whether HECT E3s contribute to such atypical ubiquitination events. Moreover, full-length HUWE1 was shown to ubiquitinate primary amines in exogenous drug-like molecules through amide linkages in cells (18), demonstrating that HECT E3s are capable of modifying synthetic small-molecule substrates. It will be interesting to investigate whether this activity extends to endogenous small molecules and which cellular pathways may be regulated by it.

### Exploiting HECT E3s therapeutically

Unlike RING E3s, HECT ligases harbor a catalytic cysteine, rendering them in principle amenable to covalent inhibition. Surprisingly, only few small-molecule screens have reported covalent active-site HECT E3 inhibitors (127, 128), whereas other efforts identified covalent binders of a non-catalytic cysteine that confers inhibition of NEDD4 by blocking the regulatory exosite (129, 130, 131). Additional, unanticipated inhibitory mechanisms of HECT E3s have emerged, including small molecules that restrict inter-lobe mobility of the HECT domain by engaging a cryptic N-lobe pocket in SMURF1 (132). Moreover, the infection-associated HECT ligase TKUL from the human pathogen *Leishmania mexicana* can be allosterically inhibited by a small-molecule kinase inhibitor, owing to its integrated regulatory kinase domain (84). Finally, the compounds BI8626 and BI8622, originally described and marketed as selective HUWE1 inhibitors (133), suppress protein ubiquitination *in vitro* through a substrate-competitive mechanism rather than direct HUWE1 inhibition, and broadly perturb ubiquitin signaling in cells (18). Together, these examples illustrate opportunities and unexpected challenges in small-molecule targeting of HECT E3s, underscoring current limitations in understanding their pharmacological susceptibilities (134). As mechanistic and structural insights expand, new strategies for HECT E3 manipulation are likely to emerge. Structural and regulatory differences across this ligase family provide multiple potential intervention points, including enzyme-specific allosteric sites, domain interfaces, and interactions with adaptors or regulators. Illuminating these features may also enable the development of HECT E3–directed targeted degraders.

In summary, structural insights into full-length HECT E3s are emerging at rapid pace. The enzymes' architectural diversity, conformational plasticity, and multifaceted specificities pose significant challenges, but underpin unique signaling capabilities and therapeutic opportunities.

## Conflict of interest

The authors declare that they have no conflicts of interest with the contents of this article.
